# Expressing Anger Is More Dangerous than Feeling Angry when Driving

**DOI:** 10.1371/journal.pone.0156948

**Published:** 2016-06-03

**Authors:** Weina Qu, Mengnuo Dai, Wenguo Zhao, Kan Zhang, Yan Ge

**Affiliations:** 1 Key Laboratory of Behavioral Science, Institute of Psychology, Chinese Academy of Sciences, Beijing, China; 2 University of Chinese Academy of Sciences, Beijing, China; Beihang University, CHINA

## Abstract

Anger is an emotion that drivers often feel and express while driving, and it is believed by researchers to be an important cause of dangerous driving behavior. In this study, the relationships between driving trait anger, driving anger expression, and dangerous driving behaviors were analyzed. The Driving Anger Scale (DAS) was used to measure driving trait anger, whereas the Driving Anger Expression (DAX) Inventory was used to measure expressions of driving anger. A sample of 38 drivers completed the DAS, DAX, and a driving simulation session on a simulator where their driving behaviors were recorded. Correlation analysis showed that the higher scores on the DAS were associated with longer durations of speeding in the simulator. The more participants expressed their anger in verbal and physical ways, the more likely they were to crash the virtual vehicle during the simulation. Regression analyses illustrated the same pattern. The findings suggest that, although trait anger is related to speeding, the passive expression of anger is the real factor underling traffic accidents. This study extends findings about the predictive effects of self-report scales of driving behaviors to behaviors recorded on a simulator. Thus, if in traffic safety propaganda, guiding drivers to use positive ways to cope with driving anger is recommended by our findings.

## Introduction

Vehicle drivers are important traffic participants. Researchers have found that driver’s properties (e.g. whether he is conservative or aggressive) significantly influenced traffic flow [1–3]. Nervous or impatient driving behaviors also had similar effect on traffic [4,5]. Dangerous drivers are different from safe ones for they have more trouble resisting negative emotional distractions[6], have higher sensation seeking level, do poorer in decision making[7], etc. More importantly, anger is one emotion that drivers often experience while driving [8]. Anger can suddenly arise in various situations and be expressed immediately [9]. Sohu [10] reported that 60.7% of respondents experienced anger when they encountered slow driving and traffic congestion while driving. As a situation-specific form of trait anger, driving anger has attracted much attention from researchers for a long time [11]. Some studies have found that drivers drive faster and more aggressively when they are angry [12–13]. Research also has found significant relationships between driving anger and dangerous driving behaviors, including losing control, fast driving, physical and verbal aggression, and traffic violations [14–16].

Several instruments have been developed to measure driving anger. Deffenbacher et al. [11] developed the Driving Anger Scale (DAS) to assess how much anger drivers experience behind the wheel. This is an instrument for assessing driving anger as a personality trait related to a general trait of anger. Measuring the level of anger is important for understanding the negative consequences of anger and its relationship to dangerous driving behavior. Several studies have investigated the situations that evoke driving anger [11,13,17,18], and a number of studies have focused on how drivers react when they are angry [19–22]. How people express their anger may be more important in a traffic situation. For example, two drivers may be equally angered by the same situation (e.g., a slow car in front of him.), but express that anger in dramatically different ways. One driver might get very angry, yell “Where did you get your license?” and give the offending driver the finger, and speed up. Another driver might merely follow the car and continue to drive safely. Therefore, how one expresses anger may play an important role in driver safety and result in different driving behaviors.

In addition to studying driving anger as a trait, it is important to study how drivers express or deal with their anger. One widely used measure of anger expression in traffic is the Driving Anger Expression Inventory (DAX) [19]. The DAX, which measures how drivers respond to anger while driving, contains four subscales: Verbal Aggressive Expression (Ver), which assesses the tendency of a driver to express anger through verbal aggression (e.g., swearing at the other driver); Personal Physical Aggression Expression (Phy), which measures the ways in which a driver uses his/her body to express anger (e.g., shaking a fist); Using the Vehicle to Express Anger (Veh), which measures how often a driver uses his/her vehicle to express anger (e.g., flashing the lights); and Adaptive/Constructive Expression, which measures constructive or adaptive behaviors a driver can perform in potential anger-inducing situations (e.g., just ignore it).

Although most studies have found a relationship between accidents and the DAX subscales, the same factors have not been consistently found. For example, one study found a significant relationship between crashes and Using the Vehicle to Express Anger [23]. However, Dahlen and Regan [24] found that accidents were positively related to the Physically Aggressive Expression subscale and negatively related to Adaptive/Constructive subscale. Perhaps, the inconsistency is related to the use of self-reported accidents in most of the studies. Measuring driving accidents in a driving simulator could overcome this limitation.

Furthermore, evidence from simulator research has found that angry drivers show more risk taking behaviors, such as driving closer to the lead car, overtaking maneuvers, and less use of the steering wheel [25,26]. These studies simply aroused a driver’s anger and measured behavioral differences in a driver’s speed and lane position. It is difficult to observe how drivers express their anger while driving (e.g., making negative comments about the other drivers or making hostile gestures). Studies of anger-driving relationships tend to focus, separately, on the influence of the anger-provoking situations or anger expression on driving behaviors; few studies have attempted to relate different driver behaviors to both factors.

Previous studies mostly focus on how anger emotion effects on driving behavior and traffic accident. As we know, anger as an emotion will last for a long time from generation, expression to extinction. However, if the effects in each stage of different anger process on driving behavior are consistent is not clear. In summary, the main purpose of the current study was to investigate the different effects of anger experiences and anger expression on driving behavior. Anger experiences were measured by the DAS, which contains 14 anger-provoking traffic scenarios; participants were asked to rate the level of anger triggered by each situation on a 5-point scale (e.g., someone is slow parking and is holding up traffic.). Anger expression was assessed by the DAX, which contains 49 potential reactions to feeling angry while driving; participants were asked to report how often they reacted with different behaviors on a 4-point scale (e.g., I bump the other driver's bumper with mine.). Driving behavior was measured by a driving simulator.

## Methods

### Participants

A total of 42 participants took part in the present study after they were told about content and possible risks of the study. Four of the participants became nauseous in the driving simulator and had to stop. The average age of the remaining 38 participants (23 males and 15 females) was 30.58 years-old (SD = 8.30). All of the participants had their driver’s license for at least 1 year, and they were all able to drive vehicles with a manual transmission. All the participants signed their informed consent before the formal experiment. They completed this experiment anonymously and voluntarily. All of their data was only used for scientific research. The participants who completed all the questionnaires and the simulated driving session received 70 yuan RMB. The Ethical approval was given by the Institutional Review Board of the Institute of Psychology, Chinese Academy of Sciences. The individual in this manuscript has given written informed consent (as outlined in PLOS consent form) to publish these case details.

### Instruments

**Fig 1:**
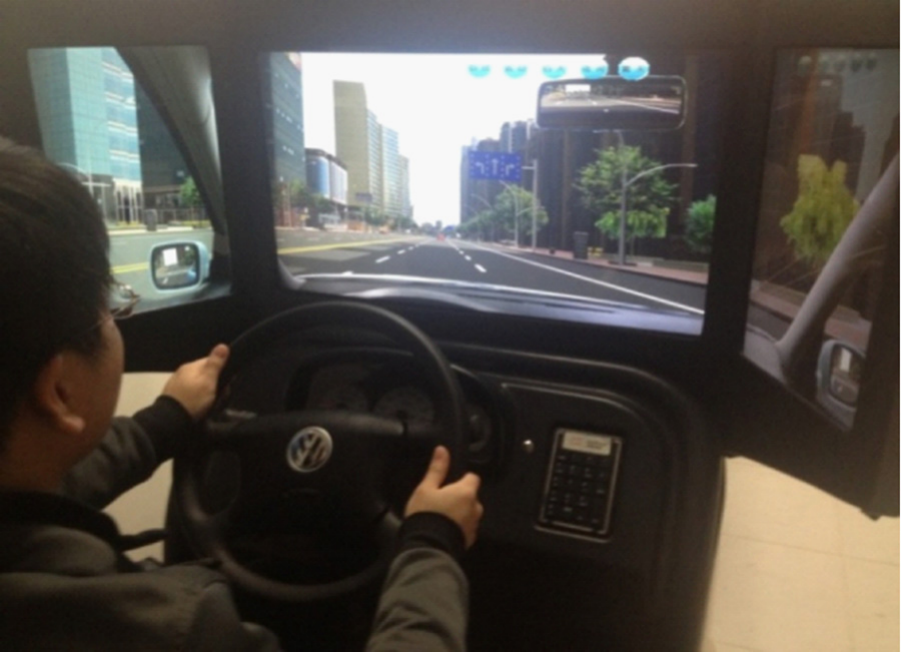
The driving simulator used in this experiment.

Driving simulator. The driving simulator (Sim-Trainer, designed by Beijing Sunheart Inc.) used in the study had an interactive cockpit, a 120° field of view, side-view mirrors, a dashboard, and a manual transmission. Participants drove by using the steering wheel, brake, and accelerator pedal[Fig 1](The participant in Fig 1 had been informed and signed agreement for this figure to be published). The driving environment was constructed by SCANeR II (developed by OKTAL of France). It is an open software platform that allows users to develop their own simulated environment easily. Using SCANeR API, we developed our own simulated driving environment with C++ language.

#### Driving environment

**Fig 2:**
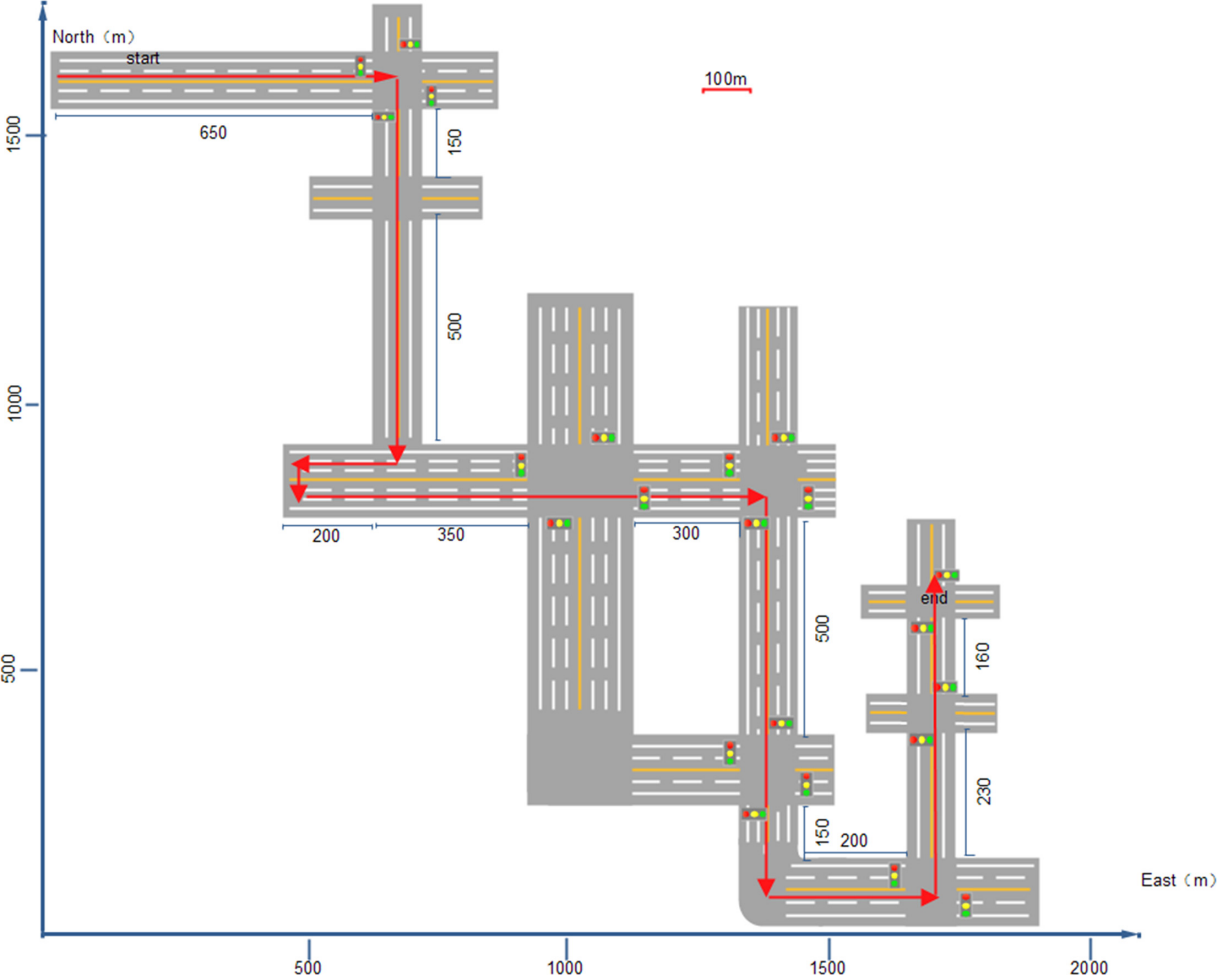
Map of the route that participants had to drive (instructed by an audio guide).

The simulated driving environment was a 3.6 km route of city streets [Fig 2]. Participants drove following audio instructions. In about one fifth of the route, the speed limit was 40 km/h, and it was 60 km/h for the rest of the route. Unexpected incidents sometimes happened. For example, a bike rider suddenly changed lanes to avoid parked cars when the participant’s car was approaching. Thus, participants should step on the brake and should not use the horn. All the driving scenarios are listed in Appendix A. The participants were told that across the whole route, speed-limit signs, traffic lights, and other traffic signs and rules worked just as they do in real life.

### Measures

#### Speed maintenance

Speed was recorded 4 times per second by the simulator across the entire route. The speed of each scenario was the average speed of the corresponding scenario. The software also recorded how long each participant exceeded the speed limit, which was saved as “duration of speeding” (in milliseconds).

#### Accidents

If the virtual car crashed with a vehicle, a bike, or a pedestrian, the image of a collision (cracks on the screen and a shrill loud sound) was presented by the simulator, and the car would be reset at the same position on the road after several seconds. The simulator recorded how many times each participant had a collision, which is the number of accidents during the simulated driving.

### Questionnaires

#### Driving Anger Scale

The Driving Anger Scale (DAS), which was developed by Deffenbacher et al.[11], is one of the most frequently used instruments for assessing trait driving anger [22]. We used the 14-item version of the DAS that was created from the 33-item Chinese version of the DAS [27]. To complete the scale, participants were instructed to imagine 14 different situations that really happened to them, and to evaluate the amount of anger they felt on a 5-point Likert scale. The final score of the scale was the average score of the 14 items.

#### Driving Anger Expression Inventory

The Driving Anger Expression Inventory (DAX) also was developed by Deffenbacher et al. [19] to measure various forms of anger expression while driving. In the present study, we used the 49-item version with 4 factors, which researchers have recommended instead of the original 53-item, 5-factor version because the fifth factor has been criticized for having low reliability.

The first 3 factors are: Verbal Aggressive Expression (Ver)–verbally expressing anger (e.g., yelling offensive words at the other driver); Personal Physical Aggression (Phy)–using the body to express anger (e.g., making hostile gestures); and Using the Vehicle to Express Anger (Veh) (e.g., driving a little faster). They cover almost all forms of the negative expressions of anger while driving. The fourth factor is the Adaptive/Constructive Expression subscale (Adp)–constructive behaviors a driver can do to cope with anger (e.g., letting go of feelings). Participants completed the four subscales using a 4-point Likert scale. The scores of the subscales are the average scores of the respective items.

#### Socio-demographic variables

Socio-demographic information was collected, including gender, age, education, weekly driving mileage, and years of driving experience.

### Procedure

First, participants freely drove on a city route, which was different from the experimental route, for 4–5 minutes to acclimate them to the simulator. After being told to obey all the traffic rules and to deal with incidents as they do in real life, participants began the experimental session of simulated driving. Then, each participant was given the questionnaires to complete. The experiment session lasted about 45 minutes.

### Data analysis

Safe driving performance was evaluated by the average speed across the entire route, duration of speeding (ms), and number of accidents. Scores of DAS’s 14 items were summed up as the DAS total score. The higher score one gets the higher level of trait driving anger he has. Scales involved in DAX are 12-item Verbal Aggressive Expression(Items 5, 6, 9, 11, 14, 28, 31, 37, 38, 39, 40 and 43), 11-item Personal Physical Aggressive Expression(Items 1, 8, 10, 12, 13, 17, 18, 20, 21, 34 and 41), 11-item Use of the Vehicle to Express Anger(Items 2, 3, 4, 7, 15, 16, 19, 22, 27, 33 and 46), 15-item Adaptive/Constructive Expression (Items 23, 24, 25, 26, 29, 30, 32, 35, 36, 42, 44, 45, 47, 48, and 49). The higher subscale score one gets, the more he tends to express his driving anger in the respective ways.

The relationships between scale scores and driving behaviors in simulator were tested with Spearman’s correlation test (2-tailed test at significant level 0.05). Later on, the predictive effect of scale scores toward driving behaviors were tested with linear regression.

## Results

### Descriptive statistics

The following Table 1 presents the descriptive statistics for all the questionnaire and simulator driving variables.

**Table 1:** Descriptive statistics for the driving-related variables.

Variable	Mean	SD	Minimum	Maximum	Median
DAS	36.026	10.789	14.000	60.000	35.000
DAX					
Ver	1.882	0.416	1.08.	2.920	1.875
Phy	1.127	0.201	1.000	1.820	1.046
Veh	1.481	0.334	1.000	2.270	1.409
Adp	2.961	0.371	2.200	3.870	2.867
Accidents	0.327	0.633	0.000	2.000	0.000
Average speed	23.359	7.193	14.360	46.971	21.371
Duration of speeding	71.493	39.891	0.000	150.250	61.375
Number of accidents	0.368	0.633	0.000	2.000	0.000

The correlations of the variables (Spearman’s rho) are presented in [Table 2] below. There were significant correlations between the DAS and the Ver, the Ver and the Veh, and the Veh and the Phy. Among the simulator driving behaviors, only average speed and duration of speeding were significantly correlated. Higher scores on the DAS were associated with longer durations of speeding in the simulator, whereas higher scores on the Ver and Phy subscales of the DAX were associated with more car accidents in the simulator [Table 2].

**Table 2:** Correlations between the driving-related variables.

	DAX	Ver	Phy	Veh	Adp	DAS	Averagespeed	Over-speed	Acci-dents
DAX									
Ver		1.000	.151	.441**	.031	.358*	-.072	.185	.580**
Phy			1.000	.341*	-.100	.168	-.036	.286	.353*
Veh				1.000	-.212	.313	.054	.280	.295
Adp					1.000	.092	.099	-.001	.027
DAS						1.000	.051	.389*	.271
Average speed							1.000	.686**	-.001
Speeding								1.000	.235
Accidents									1.000

*. p<0.05 two-tailed test

**.p<0.01 two-tailed test

### Regression

The relationships between the scale scores and driving performance were tested using linear regression. The numbers of simulator accidents and the duration of speeding were predicted by the respective scale scores with which they were correlated.

#### Accidents

According to regression analyses, the models that included Ver and Phy scores significantly predicted the number of simulator accidents (*F* = 7.881, *p* = 0.001). The coefficient of determination (*r²*) was 0.311. The coefficients (as in the 2-variable model below) for both scale scores were positive. The more participants tended to express driving anger verbally or physically, the more accidents they had in the simulator.

Accidents=0.409×Ver+0.282×Phy−1.806

#### Speeding

For speeding, on the other hand, the model that included the DAS score proved to be statistically significant (*F* = 5.537, *p* = 0.024, *r²* = 0.133). The coefficient was positive, as well, which means that higher trait driving anger predicted longer durations of speeding in the simulator.

Over−speed=0.365*DAS+22861.307

## Discussion

### Summary of the findings

The main objective of this research was to investigate whether there are different effects between driving anger and the expression of driving anger on dangerous driving behavior. Overall, drivers who felt anger that was triggered by specific anger situations only exhibited speeding behavior. However, the way they expressed their anger was more dangerous and could cause an accident.

Driving anger induced by anger-provoking situations had a significant correlation with speeding but no correlation with the number of accidents. This result is consistent with previous studies. Anger-prone drivers have reported driving faster and failing to comply with the speed limit [19,28]. Deffenbacher et al.[29] reported that anger is a personality characteristic that makes people prone to be angry while driving, which is not directly related to having an accident. Drivers who had more anger while driving reported having a higher number of near-miss accident [8]. This study measured anger-provoking scenarios that drivers may encounter while driving (e.g. someone speeds up when you trying to pass them). According to Matthews [30], inherently angry drivers are more inclined to make hostile evaluations of traffic situation and, consequently, drive faster. Our study demonstrated that drivers who were more easily enraged by driving situations were more likely to speed. However, it does not provide evidence that proves they were more likely to be involved in an accident.

Nevertheless, our research found that Verbal Aggressive Expression and Personal Physical Aggression Expression had significant correlations with the number of accidents. There are many studies that have identified a relationship between the DAX subscales and crashes. Verbal Aggressive Expression has been found to be related to loss of concentration, loss of control, near-misses, and crashes [18,19,31]. Previous studies also found a correlation between Personal Physical Aggression and accidents. Deffenbacher et al. [19] found Personal Physical Aggression was related to traffic tickets, but not to crashes,. However, Sullman et al. [15] found no relationship between Personal Physical Aggression and traffic tickets, but significant relationships with loss of concentration, loss of control, and near-misses. A Malaysian study found Personal Physical Aggression was related to traffic tickets, loss of control and crashes [16]. Most previous research studied self-reported accidents. In the current study, we measured accidents using a driving simulator and found that Personal Physical Aggression had a positive significant correlation with accidents.

### Limitations

This research has a number of limitations. First, driving anger and and the expression of angry driving were measured by self-report data. These anger variables could not measure online nor could we measure the actions of the drivers during the simulated driving session. A better approach would be to design driving situations using a driving simulator that elicits anger and to measure related driving behavior. Future studies should explore this possibility, which would improve the ecological validity of these studies. Second, driving behavior was measured by the driving simulator, which is likely to have some degree of artificiality. If we were able to obtain actual traffic records, we could analyze how driving anger and its expression affect real driving behavior. Further research should pay more attention to field observation and actual traffic records.

### Implications

The current study investigated the different effects of anger experiences and anger expression on driving behavior. Result demonstrated expressing anger is more dangerous than feeling angry when driving. Therefore, if in traffic safety propaganda, guiding drivers to use positive ways to cope with driving anger is recommended by our findings. This study also has important practical implications for traffic safety. For example, the results of this study can be used to design training classes that provide some useful ways to relieve angry emotion. Such programs would improve drivers’ emotional cognition and help them to modify their dangerous driving behavior.

## Supporting Information

S1 File Behavior data for all participants. Behavior data for all participants.
(XLSX)

S2 File **Appendix A.** Description of all of the scenarios in simulating procedure.
(DOCX)
