# Supplementary material for: Expressing Anger Is More Dangerous than Feeling Angry when Driving
Source: PLoS One. 2016 Jun 3;11(6):e0156948. doi: 10.1371/journal.pone.0156948 (PMC4892685; doi:10.1371/journal.pone.0156948)
Supplement: S2 File — Appendix A. Description of all of the scenarios in simulating procedure. (DOCX) [file pone.0156948.s002.docx]

## Appendix A

**Description of the scenarios**

| Number | Event’s description |
| --- | --- |
| 1 | The cars following the participant’s car are blowing their horns. |
| 2 | When the voice prompts "turn right at the next intersection", five pedestrian are walking along the crosswalk. |
| 3 | A bicycle is running in front of the car. |
| 4 | The car is entering the 1km-long two-lane two-way tunnel, the light suddenly dimmed, where the speed limit 40km / h. |
| 5 | In a tunnel, a vehicle is suddenly overtaking in the opposite direction (an illegal behavior), there is a possibility of collision. |
| 6 | A truck drove slowly in front of the participant’s car. |
| 7 | There is no car around a two-way four-lane road, a student is walking across the road. |
| 8 | The car runs into the uphill roads where traffic jams. |
